# Diagnosis and Management of Progressive Multiple Sclerosis

**DOI:** 10.3390/biomedicines7030056

**Published:** 2019-07-29

**Authors:** Gabrielle Macaron, Daniel Ontaneda

**Affiliations:** Mellen Center for Multiple Sclerosis, Neurological Institute, Cleveland Clinic Foundation, Cleveland, OH 44195, USA

**Keywords:** progressive multiple sclerosis, neurodegeneration, remyelination, outcome measures, biomarkers

## Abstract

Multiple sclerosis is a chronic autoimmune disease of the central nervous system that results in varying degrees of disability. Progressive multiple sclerosis, characterized by a steady increase in neurological disability independently of relapses, can occur from onset (primary progressive) or after a relapsing–remitting course (secondary progressive). As opposed to active inflammation seen in the relapsing–remitting phases of the disease, the gradual worsening of disability in progressive multiple sclerosis results from complex immune mechanisms and neurodegeneration. A few anti-inflammatory disease-modifying therapies with a modest but significant effect on measures of disease progression have been approved for the treatment of progressive multiple sclerosis. The treatment effect of anti-inflammatory agents is particularly observed in the subgroup of patients with younger age and evidence of disease activity. For this reason, a significant effort is underway to develop molecules with the potential to induce myelin repair or halt the degenerative process. Appropriate trial methodology and the development of clinically meaningful disability outcome measures along with imaging and biological biomarkers of progression have a significant impact on the ability to measure the efficacy of potential medications that may reverse disease progression. In this issue, we will review current evidence on the physiopathology, diagnosis, measurement of disability, and treatment of progressive multiple sclerosis.

## 1. Introduction

Multiple sclerosis (MS) is a chronic inflammatory disease of the central nervous system that affects over 2.3 million people globally, with an estimated prevalence of approximately 310 per 100,000 population in the United States [1,2]. Most patients (~90%) have relapsing–remitting disease at onset, which typically is followed by a secondary progressive course, while a minority of patients have a primary progressive course from onset (~10%). Relapsing–remitting MS (RRMS) is characterized by frequent formation of inflammatory lesions in the brain and spinal cord. Approved disease-modifying therapies (DMTs) target the inflammatory component of the disease, and strong evidence support their effectiveness in RRMS. However, trials evaluating their efficacy in slowing disease progression have shown mixed results, or have shown only modest effects in slowing progression. The goal of this review is to provide a comprehensive overview on the current knowledge of the pathogenesis, diagnosis, and treatment of progressive MS, as well as future directions in the field.

## 2. Pathogenesis

The pathogenesis of MS is incompletely elucidated. This is particularly the case for progressive MS, for which various and sometimes conflicting data have been proposed to explain the underlying pathogenic process of progression [3]. In RRMS, actively demyelinating plaques are the most prominent lesion type, and are characterized by inflammatory demyelination and axonal transection within the lesions [4,5,6]. However, active lesions are rare in progressive MS, and axonal transection is not seen as frequently within inactive lesions compared to highly inflammatory recently developed lesions [3,4,5]. Whole brain atrophy, smoldering and enlarging lesions, cortical demyelination (specifically subpial lesions), and diffuse axonal injury and microglial activation in normal appearing grey and white matter are prominent in patients with progressive MS compared to patients with early RRMS [6,7,8,9,10]. Disability in progressive MS is thought to be related to secondary neurodegeneration of chronically demyelinating axons, which is thought to be driven by a series of factors, including: (1) inflammation and lesion accumulation, with subsequent retrograde and anterograde degeneration, (2) mitochondrial damage and subsequently virtual hypoxia and oxidative stress, (3) iron accumulation in myelin sheath and oligodendrocytes with subsequent amplification of oxidative stress, (4) lymphoid follicle-like structures that might contribute to sustaining cortical pathology, and [5] age-related neurodegeneration and reduced neuronal reserve (loss of the ability to compensate for axonal loss) [3,11,12,13,14,15]. A recent paper by Brown et al. [16], among others, showed that the early use of DMTs, specifically highly-effective DMTs, decreases the odds of conversion to secondary progressive MS (SPMS), which supports the role of early disease activity in the development of long-term disability progression [16,17,18,19,20,21]. The role of age-related mechanisms is supported by the fact that children with MS rarely present with progressive disease and have a longer time to reach secondary progression and disability milestones compared to adult-onset MS, and that certain disability milestones are acquired at certain ages independently of the duration of the disease [22,23]. However, for primary progressive MS (PPMS), the time course of irreversible damage is not clearly affected by the presence or absence of superimposed relapses [24]. Recent observations support a change in the natural history of MS with earlier use of highly-effective DMTs; however, this effect seems to be more clearly evidenced in RRMS [25]. Despite the wide variability of clinical and radiological presentations and the inherent pathological differences between RRMS, SPMS, and PPMS, the consensus is that PPMS is biologically part of the MS spectrum [13].

## 3. Diagnostic Criteria and Disease Course Definitions

**Figure 1:**
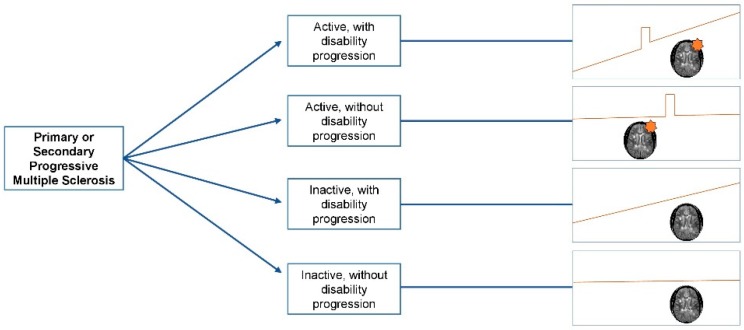
Clinical course of progressive multiple sclerosis. The orange star indicates the presence of radiological activity (new/enlarging T2 lesions or gadolinium enhancing lesions).

The diagnosis of progressive MS is based on patient-reported clinical history, and should be confirmed based on objective physical examination findings. Based on the 2017 McDonald diagnostic criteria, PPMS can be diagnosed in patients with a 1-year history of disability progression, which can be retrospectively or prospectively determined, independent of clinical relapses, plus two of the following criteria: (1) One or more T2 lesions characteristic of MS in one or more typical brain regions (periventricular, cortical or juxtacortical, infratentorial); (2) two or more T2 lesions in the spinal cord, and (3) the presence of CSF-specific oligoclonal bands. Unlike the 2010 McDonald criteria, both symptomatic and asymptomatic MRI lesions are taken into account [26,27]. The panel also recommends specifying a provisional disease course at the time of diagnosis, and whether disease activity and/or progression are present or not based on the previous year’s history, which can then be revisited based on periodic re-evaluation [27]. Providing a clinical definition of disease progression, however, is somewhat harder. Progression is characterized by a steady increase in neurological disability occurring independently of relapses [27,28]. Symptoms can fluctuate (i.e., pseudo relapses), and bona fide superimposed relapses might occur. Detailed history taking is key in differentiating events suggestive of disease activity from worsening of previously experienced symptoms in the context of fatigue, heat, or stress. PPMS is defined by a progressive course from onset and SPMS by a progressive course following an initial relapsing–remitting course. The 2013 revisions of MS clinical course definitions aimed at standardizing the terminology across clinicians and researchers, for prognostication, design of clinical trials, and treatment decisions purposes [28]. These definitions included the presence or absence of clinical or radiological activity, and the presence or absence of disability progression into the phenotypic description of the disease. Figure 1 illustrates the currently used description of different progressive MS phenotypes. The distinction between “active” and “inactive” progressive disease, whether primary or secondary, has important therapeutic implications. This will be discussed in the treatment section of this manuscript.

## 4. Disability Outcome Measures

Disability progression in MS affects multiple functional domains, occurs insidiously over time, and can be difficult to quantify in an objective, comprehensive, and reproducible manner. Reliable detection of clinical and sub-clinical progression is key to interpreting treatment efficacy in trials and in clinical practice of DMT and repair promoting strategies in progressive MS. The expanded disability status scale (EDSS) is the most commonly used clinical outcome measure in trials for quantification of physical disability in MS. However, the EDSS does not comprehensively reflect disability status, and is particularly restricted in assessing cognitive and upper extremity functions [29]. Other limitations of the EDSS include poor intra and inter-rater variability especially for lower scores. The test also shows little sensitivity to detect change, especially in patients with scores of 6.0 or more, and the EDSS is difficult to administer in routine care [30,31,32]. Since it is an ordinal scale, changes in EDSS scores are not equivalent across the range of the scale. Finally, the most commonly used outcome measure in progressive MS trials is the 3 or 6-month confirmed disability progression, which might be insufficient to predict long-term disability worsening [33]. Evaluating multiple functional domains improves the likelihood of observing a change in patients with MS. The multiple sclerosis functional composite (MSFC) [34,35] was developed as a quantitative clinical measure of neurologic disability to overcome some of the shortcomings of the EDSS. Cognitive function was evaluated originally using the paced auditory serial addition test (PASAT), but more recently using the symbol–digit modalities test (SDMT), and upper extremity function is evaluated using the 9-hole peg test (9HPT) [36,37]. Walking speed is usually evaluated using the timed-25-foot walk test (T25FW). The MSFC has proven to be more sensitive to change than the EDSS, and correlates with subsequent changes in EDSS [34] T1 and T2 lesion load on brain MRI [38], and patient reported physical and emotional functioning and quality of life [39]. In the interferon beta (IFN-β)-1a SPMS trial, there was some benefit of treatment on the MSFC z-scores but not on the EDSS [40]. This benefit was mainly driven by two of the components of the MSFC, the 9HPT and the PASAT, which further illustrates the importance of a comprehensive neurological evaluation to assess disability progression. The low contrast letter acuity testing using a low-contrast Sloan letter chart was later proposed as an additional component of the MSFC to capture visual dysfunction with a high sensitivity, and also correlates with other components of the MSFC and the EDSS [37,41,42]. In addition, internal consistency (reliability) is higher for the MSFC (Cronbach’s alpha coefficient 0.93−0.96) than for the EDSS (Cronbach’s alpha 0.7) [43,44]. Although the MSFC has been used as an outcome measure in clinical trials [40,45,46], administration in routine clinical practice is time consuming, and requires personnel to administer the test properly [47]. Recently, there has been increasing interest in developing tools to facilitate and standardize testing in MS patients. For example, a technology-enabled version of the MSFC has been developed and was incorporated into routine clinical practice. This tool, called the multiple sclerosis performance test (MSPT), comprises a battery of quantitative neuroperformance assessments administered using a suite of iPad^®^ applications modeled after the MSFC approach, and has allowed the gathering of large-scale comprehensive and standardized measures of disability from routine care [48,49]. A limitation of these outcome measures is the uncertainty of what constitutes a clinically meaningful change, although a threshold of ± 20% change in T25FW [50,51] ±15–20% in the 9HPT [52,53] ±10% (or 4 points) in the SDMT [53,54] and ±7 letters in the low contrast letter acuity test [55] have been suggested as clinically meaningful in MS.

## 5. Measuring Disease Progression

Numerous imaging biomarkers that have been proposed for the monitoring of progressive disease in MS trials, whole brain atrophy being the most widely used. Monitoring of T1 hypointensity evolution over time on conventional imaging has also been suggested. Other more advanced MRI techniques that can reflect axonal loss in progressive MS include thalamic volume, spinal cord atrophy, hippocampal volume, gray matter fraction, cortical lesion quantification, and sodium imaging, among others [56,57]. Several additional advanced MRI measures have been used as exploratory outcomes [58] as well as neurophysiological measures, such as evoked potentials [59]. Optical coherence tomography (OCT) is a non-invasive tool that allows the measurements of retinal nerve fiber layer thickness, ganglion cell/inner plexiform layer thickness, and macular volume. Since these measurements correlate with whole brain and gray matter atrophy and physical disability, OCT can serve as an outcome measure of axonal loss in phase 2 proof of concept clinical trials of progressive MS [60]. Other imaging and non-imaging techniques are used in remyelination trials as biomarkers of myelin repair, and include magnetization transfer ratio, diffusion-weighted imaging, myelin water imaging, and visual evoked potentials [56].

There has been increasing interest in the use of serum neurofilament light chain (NfL) as a biomarker in MS over the past few years [61]. Serum NfL levels correlate with CSF NfL levels and reflect disease activity and response to therapy [61]. In progressive MS, NfL levels appear to be associated with superimposed clinical or radiological activity, as well as T1-hypointense lesion volume [62]. Recent data suggests that CSF NfL correlates with disease activity rather than progression, irrespective of the disease subtype, and does not reflect disease severity [63,64], whereas previous data report a strong correlation between NfL levels and future worsening on the EDSS and brain and cervical spinal volume loss [65,66]. Serum NfL levels can be easily obtained, and are being further investigated and used as an outcome measure in newer progressive MS trials.

## 6. Treatment

### 6.1. Anti-Inflammatory Disease-Modifying Therapies

#### 6.1.1. Approved Therapies

Ocrelizumab (*Ocrevus^®^*) a humanized monoclonal antibody targeting the CD20 antigen on B-cells. Ocrelizumab exerts its anti-inflammatory effects by causing rapid and profound depletion of B cells. Ocrelizumab has been studied in patients with RRMS in two phase 3 double-blind, placebo-controlled randomized clinical trial (OPERA I and II) [67] and in patients with PPMS (ORATORIO trial) [68], but not in patients with SPMS. Participants with PPMS were required to have positive oligoclonal bands to enroll in the study. In the ORATORIO trial ocrelizumab significantly reduced the risk of 24-week confirmed disability progression compared to placebo (29.6% with ocrelizumab versus 35.7% with placebo). Treatment with ocrelizumab in PPMS also decreased worsening on the timed-25-foot walk, T2 lesion volume, and brain atrophy at 120 weeks compared to placebo. In a pre-specified subgroup analysis, the magnitude of the effect of ocrelizumab was larger in patients with baseline enhancing lesions and younger patients; however, older patients without enhancing lesions at baseline also derived benefit across primary and secondary endpoints [68]. The ENCORE study evaluated the effect of ocrelizumab on upper limb function in the ORATORIO cohort, and also showed positive results (reduction in time—to 12- and 24-week confirmed ≥15% increase on 9HPT by 37% (hazard ratio 0.627; *p* = 0.001) and 39% (HR = 0.607; *p* = 0.002) for both-hands) [69]. Ocrelizumab was approved by the FDA and the EMA to treat PPMS in 2017. Ongoing trials aim at evaluating the effect of ocrelizumab on hand function in patients with more advanced disability (ORATORIO-HAND, NCT03562975), and in a broader range of patients (with PPMS and SPMS, up to 65 years old) (CONSONNANCE, NCT03523858). It is important to note that previous trials using the chimeric monoclonal antibody rituximab, which has a similar mechanism of action as ocrelizumab, yielded results in one pivotal trial that shaped the inclusion criteria for the ORATORIO study. In the OLYMPUS trial of rituximab, the primary endpoint was negative but sub-group analysis found that younger patients with clinical or radiological evidence of disease activity did derive treatment benefit. The subgroup of patients who were older and did not have gadolinium-enhancing lesions had faster disability progression than those on placebo [70]. This indicates a potential harm of treating with a B-cell therapy in this population. Another recent observational study using retrospective data from three European centers and propensity score matching, analyzed the effect of rituximab treatment on disability progression in patients with SPMS. In this study, patients with SPMS treated with rituximab had significantly delayed confirmed progression compared with matched untreated controls at up to 10 years [71]. The difference in effects seen between ocrelizumab and rituximab are most likely related to trial design; however, biological differences may exist as well, given that ocrelizumab appears to promote cell death via greater antibody-dependent cellular cytotoxicity (ADCC) activity and less complement-dependent cytotoxicity (CDC) activity compared to rituximab, and has a more favorable antigenic profile compared to rituximab [72,73,74].

Siponimod (*Mayzent^®^*) is a selective sphingosine-1-phosphate receptor 1 and 5 modulator, which inhibits the egress of lymphocytes from lymph nodes, thus decreasing their entry into the CNS. In addition to its anti-inflammatory effects, siponimod has been found to have putative neuroprotective and repair properties in preclinical studies. It was recently approved for the treatment of SPMS based on the results of the EXPAND trial [75]. Compared to the placebo group, a significant reduction in time to 3 and 6-month confirmed disability progression (relative risk reductions of 21% and 26%, respectively) was observed in the siponimod group, and this trend was consistent in subgroup analysis with respect to pre-treatment relapse activity, disease progression rate, and disease severity. Significant reduction in markers of disease activity were also observed in the siponimod group, including annualized relapse rate, time to relapse, and gadolinium-enhancing lesions, and new/enlarging T2 lesions. Brain volume change from baseline was lower in the siponimod group at both months 12 and 24 compared to placebo [75]. Pre-clinical data also suggest that sphingosine-1-phosphate modulators cross the blood–brain barrier, and have the potential to improve morphological markers of remyelination [76]. In addition, modulation of sphingosine-1-phosphate receptor 5 has been shown to promote remyelination in vitro [76]. Interestingly, fingolimod, a sphingosine-1-phosphate receptor 1 to 5 modulator, failed to show significant reduction in confirmed disability worsening in patients with PPMS in the INFORMS trial [77]. The fact that other DMTs with a similar mechanism of action to siponimod and ocrelizumab failed to show benefit in PMS can be due to different patient population and sub-optimal trial designs, but also illustrates the lack of efficacy of anti-inflammatory strategies in the prevention of disability worsening in inactive PMS, and the need to develop molecules with a potential effect on neurodegeneration.

Mitoxantrone is a DNA intercalating agent that interferes with the replication and proliferation of B and T lymphocytes. Its use is nowadays limited due to the well-known serious adverse events (including cardio-toxicity, leukemia, amenorrhea, infections, alopecia, leucopenia, anemia, and hepatotoxicity) [78], and the availability of safer DMTs. The mitoxantrone in progressive multiple sclerosis (MIMS) trial was a double-blind, multicenter, phase 3 trial that randomized patients with worsening RRMS or SPMS to placebo or low (5 mg/m^2^) or high (12 mg/m^2^) dose mitoxantrone for 2 years [79]. About half of the participants had SPMS, with or without clinical activity in the year prior to enrollment. The primary outcome was a combination of five clinical measures: change from baseline EDSS at 24 months, change from baseline ambulation index at 24 months, number of relapses treated with corticosteroids, time to first treated relapse, and change from baseline standardized neurological status at 24 months [79]. In the cohort as a whole, a beneficial effect on the primary outcome clinical composite measure was observed for the mitoxantrone, with comparable treatment effects in patients with and without relapses in the year prior to enrollment. A few years later, the MIMS trial group analyzed the effect of low and high-dose mitoxantrone on measures of radiological activity in a subgroup of patients with worsening RRMS or SPMS, and surprisingly did not show a consistent effect of mitoxantrone on the presence of gadolinium-enhancing lesions for up to 24 months compared to placebo [80]. Mitoxantrone is approved by the FDA for the treatment of RRMS, SPMS, and what was previously referred to as “progressive relapsing MS”. There is no evidence that supports a benefit of mitoxantrone in PPMS without clinical or radiological activity [81,82]. Single-nucleotide polymorphism in the ATP-binding cassette transporter genes may serve as pharmacogenetic markers associated with clinical response to mitoxantrone in RRMS and SPMS [83]; however, this association was not observed in patients with PPMS [81].

Cladibine produces rapid and long-lasting reductions in T-lymphocytes and rapid but transient reduction in B-lymphocytes, by disrupting cellular metabolism, inhibiting DNA synthesis and repair, and subsequent apoptosis of affected cells. The oral formulation of cladribine (*Mavenclad^®^*) has recently received FDA approval for the treatment of active RRMS and SPMS based on the results of the CLARITY and ORACLE MS trials, and post-hoc analysis of the ONWARD trial [84,85,86,87]. Intravenous formulations of cladribine have been mainly studied in progressive MS. An initial small trial (24 matched patients with clinically definite progressive MS as defined at the time of the study, baseline EDSS of 4.7, randomized to receive IV cladribine or placebo) showed a significant benefit of cladribine on EDSS worsening, with some patients even experiencing improvement on EDSS at month 12 [88]. There was also a positive effect on T2 lesion volume in this study. Another larger trial was then conducted in light of these results. Patients with SPMS or PPMS and a median baseline EDSS score of 6.0 were randomly assigned to receive either placebo or cladribine 0.07 mg/kg/day for 5 consecutive days every 4 weeks for 2 or 6 cycles, followed by placebo, for 8 cycles [89]. No benefit of cladribine on the primary outcome (mean change in EDSS at month 12) was observed compared to placebo. As expected, there was a significant effect of cladribine on gadolinium-enhancing lesions and T2 lesion accumulation, and a somewhat marginal benefit in a subgroup analysis of SPMS patients. Similarly, in another study, cladribine did not have a beneficial effect on whole brain volumes compared to placebo [90].

In the ONWARD trial, the effect of oral cladribine as an add-on to IFN-β in patients with active RRMS or SPMS was studied [87]. As expected, cladribine + IFN-β was superior to placebo + IFN-β in reducing annualized relapse rate and gadolinium-enhancing lesions. However, the confirmed EDSS progression over 96 weeks was similar between the two groups. In a post-hoc analysis of subgroups in the intention-to-treat population, cladribine + IFN-β was superior to placebo + IFN-β in reduction of annualized relapse rate (relative risk ratio of 0.11, 95% CI 0.01–0.94) in patients with active SPMS. Hence, oral cladribine was approved by the FDA for the treatment of active SPMS, but not for progressive MS without evidence of clinical or radiological activity.

#### 6.1.2. Therapies with Negative or Weak Effect in Progressive MS

Studies of other anti-inflammatory DMTs in progressive MS have yielded deceiving results. Interferon-beta (IFN-β) has complex immunomodulatory effects (downregulation of pro-inflammatory and upregulation of anti-inflammatory cytokines). There is no robust evidence of the beneficial effect of IFN-β on progression, although early treatment in RRMS decreases conversion to SPMS, which is attributable to its anti-inflammatory properties [16,17,18,19]. In SPMS, IFN-β significantly delayed time to confirmed disability progression compared to those receiving placebo in the European SPMS IFN-β-1b trial [91]. However, discrepant results were observed in the North American SPMS IFN-β-1b trial, which did not show a difference in time to 6-month sustained EDSS progression compared to placebo [92]. A post hoc pooled analysis of the clinical trial data of both groups was performed to better clarify this discrepancy, and showed that patients in the European studies who benefited from treatment were significantly younger (41 vs. 46.9 years, *p* < 0.001), with shorter disease duration (13.1 vs. 14.7 years, *p* < 0.001), and more active disease (number of relapses in the last 2 years 1.7 vs. 0.8, contrast-enhancing lesions 2.6 vs. 1.5, *p* < 0.001) [93]. The lack of benefit of IFN-β-1b in patients with SPMS with less active disease was confirmed in other SPMS trials as well [94,95].

IFN-β trials in PPMS have also been largely mixed, with negative results on primary (confirmed disability progression) and most secondary endpoints [96,97,98], although significant differences in MSFC scores, MRI T2 lesion volume, and MRI T1 lesion volume after 2 years of treatment favoring IFN-β-1a was observed in one trial [98].

Glatiramer acetate (GA), a synthetic polypeptide with a complex and incompletely understood immunomodulatory mechanism of action, was studied in patients with PPMS in the PROMiSe trial, a multicenter, placebo-controlled, double-blind randomized clinical trial comparing GA to placebo over a 3-year period [99]. This trial was successful in including a large majority of patients without signs of disease activity. GA decreased markers of radiological activity (gadolinium enhancing lesions and accumulation of T2 lesions), and had some benefit on disability progression in males, but there was no effect on the primary outcome and the study was terminated early [99,100].

As discussed earlier in this manuscript, the INFORMS trial failed to show a positive effect of fingolimod (*Gilenya^®^*) on reduction in confirmed disability worsening in patients with PPMS [77]. The primary endpoint was defined by a composite of outcomes including the EDSS, T25FW, and 9HPT. There was no benefit of fingolimod on the disability composite (HR = 0.95, 95%FW CI 0.80–1.10, *p* = 0.544). Fingolimod has not been studied in SPMS.

Natalizumab (*Tysabri^®^*) is a monoclonal antibody that exerts its potent anti-inflammatory effect by inhibiting the alpha-4 integrin and subsequently preventing the migration of T-lymphocytes across the blood–brain barrier. The ASCEND trial evaluated the effect of natalizumab on a composite score including the EDSS, T25FW, and 9HPT in patients with SPMS [101]. No benefit was observed on the composite primary outcome, and individually on the EDSS and 9HPT, although in a post hoc analysis, there was a 44% reduction in hand function progression measured by the 9HPT (OR 0.56, 95% CI 0.40–0.80, *p* = 0.001).

Rituximab is a chimeric monoclonal antibody targeting the CD20 antigen on pre-B-cells and mature B-cells that has been used in many autoimmune neurological disorders of the central and peripheral nervous systems for decades [102]. Similarly to ocrelizumab, it causes rapid and profound depletion of B-cells via antibody-dependent cellular cytotoxicity (ADCC) and complement-dependent cytotoxicity (CDC) mechanisms leading to B-cell death [73,74]. Rituximab has been used off-label to treat progressive MS in certain countries, and there has been long-standing evidence of its efficacy to control inflammatory disease activity in observational studies [103]. Furthermore, as discussed above, a recent study also suggests that rituximab significantly delayed confirmed progression in SPMS [71]. The OLYMPUS trial evaluated the effect of rituximab on disability progression in patients with PPMS [70]. This was a phase 2/3 multicenter, placebo-controlled trial involving 439 patients with PPMS for 96 weeks. There were no differences in the primary endpoint in the overall cohort (time to 12-week confirmed disability progression using the EDSS at 96 weeks). There was a significant effect on T2 lesion volume, which was lower with rituximab. An important point of this trial is the result of the subgroup analysis, which showed a significant difference on the primary endpoint in the subgroup of patients who were <51 years old and who had baseline enhancing lesions (hazard ratio 0.52 (*p* = 0.010) and 0.41 (*p* = 0.007), respectively), whereas rituximab-treated patients who were older than 51 years and had no enhancing lesions at baseline had non-significant but worse outcomes than the placebo group (hazard ratio 1.27 (*p* = 0.425)). This highlights the predominantly anti-inflammatory effect of B-cell therapies in MS.

There has been evidence of the presence of lymphoid follicle-like structures in the cerebral meninges that are typically adjacent to large subpial lesions, and associated with more severe cortical pathology and accelerated disability progression in patients with SPMS [14,15]. Based on these observations, the eventual role of these lymphoid follicles in sustaining cortical injury and accelerating clinical worsening was hypothesized, and the effect of intrathecal rituximab was evaluated since IV rituximab does not cross the blood–brain barrier [104,105,106,107]. The RIVITALISE trial, a randomized, double-blind trial study of intravenous and intrathecal rituximab in patients with SPMS, showed that intrathecal rituximab transiently decreased the B cell counts in the CSF and did not induce consistent effects on CSF biomarkers [104].

A very recent trial evaluated the safety and efficacy of intrathecal rituximab in eight patients with progressive MS who had focal leptomeningeal contrast-enhancement on contrast-enhanced T2-FLAIR [105]. Transient reduction in CSF B cells and biomarkers (reduction in chemokine ligand 13 (CXCL-13) levels with an increase in B cell-activating factor belonging to the TNF family (BAFF) levels), along with profound peripheral B cell depletion was observed; however, the number of leptomeningeal lesions did not change.

Other immunomodulating and immunosuppressive therapies have been investigated in PMS. Examples include azathioprine [108,109,110], cyclophosphamide [111,112,113,114], intravenous immunoglobulins [110,115], methotrexate [116,117,118], cyclosporine [109,119], mycophenolate mofetil [120], laquinimod [121], and MBP8298 [122]. Results from these trials have been largely negative, with no or modest benefit on markers of disability progression.

### 6.2. Remyelination and Neuroprotection in Progressive MS

While some agents have shown to be beneficial on slowing disability progression, no molecules have shown to have an effect on halting progression or reversing neurological damage in well-powered clinical trials. There are two therapeutic approaches that are thought to be promising to achieve the latter: remyelination and neuroprotection. Remyelinating agents can theoretically repair damage and neuroprotective agents can theoretically prevent axonal loss. High-throughput methods have generated many promising remyelinating molecules to test in pre-clinical studies, to be followed by phase 1, 2, and 3 trials. Unfortunately, studies have been mostly negative or have shown only modest benefits on measures of brain atrophy for which clinical significance still needs to be better elucidated. Table 1 provides an overview of positive trials of molecules with a putative remyelinating and neuroprotective effect. Some agents such as biotin and mesenchymal stem cells may have both remyelinating and neuroprotective effects; these medications are discussed under neuroprotection.

In MS, progression secondary to neurodegeneration is thought to be secondary to chronic demyelination [3]. While neuronal cell bodies and axons have a low potential of regeneration, myelin may have a potential for repair. Remyelination can be seen in shadow plaques, which supports this potential repair property likely mediated by oligodendrocytes [3,6]. A few compounds promoting endogenous oligodendrocyte progenitor cell differentiation have been found in pre-clinical work to be promising remyelinating agents [156]. Out of thousands of compounds identified through high-volume screening of existing drugs [124], opicinumab [128,129,130], and clemastine [125] have been studied in phase 2 trials. Clemastine demonstrated improvement in P100 latency on visual evoked potential in patients with chronic optic neuropathy compared to placebo [125]. Opicinumab showed a similar effect in patients with acute optic neuritis [128,129] (Table 1). Other potentially effective molecules are currently being evaluated, including domperidone (in SPMS, NCT02308137), quetiapine (in RRMS and progressive MS, NCT02087631), liothyronine (in RRMS and progressive MS, NCT02760056), among others. Miconazole and clobetazol have recently been identified as agents with a potential to produce mature oligodendrocytes from progenitor cells [157].

Most of the trials of molecules with a potential for axonal repair have been deceiving. The SPRINT-MS study results were promising; however, phase 3 trials are needed to confirm this result before considering ibudilast as a therapy for progressive MS [132]. Similarly, lipoic acid and simvastatin [134] appear to be potentially beneficial in slowing brain atrophy, and further studies are needed to confirm the results of the phase 2 trials. In a randomized placebo-controlled phase 2 trial, phenytoin was found to have neuroprotective effects after acute optic neuritis compared to placebo [139]. The MS-SMART trial (NCT01910259) failed to show a benefit of amiloride, fluoxetine, or riluzole on brain atrophy in progressive MS (results presented at ECTRIMS 2018). High-dose biotin [153,154,155] and mesenchymal stem cells appear to have a dual effect on neuroprotection and remyelination, and have been studied in multiple small trials and open-label studies in progressive MS, with promising results (Table 1). It is important to note that treatment with autologous hematopoietic stem cell transplantation is reserved for patients with treatment-refractory highly active MS, specifically those who have poor prognostic factors of future disability, including ongoing clinical or radiological activity despite treatment with potent DMT [158], and is out of the scope of this review. Other compounds that are being investigated in phase 2 or 3 trials, include idebenone (NCT01854359), masitinib (NCT01433497), hormone-based therapies (ACTH (NCT01950234)), and erythropoietin (NCT01144117), lithium (NCT01259388), and T-cell receptor vaccines (NCT02057159), among others.

### 6.3. Symptomatic Management

Optimal symptom management is essential to improve quality of life of patients and to complement the beneficial effect of long-term maintenance therapies in MS. The most commonly encountered symptoms in MS include fatigue, spasticity-related symptoms, neuropathic pain, urinary dysfunction, sleep disturbances, and mood changes. A high number of patients complain of more than one symptom, many of which may be interrelated. For example, poor sleep and depression worsens diurnal fatigue, which can hence not solely be attributed to the disease. Routine evaluations should include screening for persistent symptoms, preferably using validated scales. A general rule in our experience is to start by treating the most disabling or consequential symptom, titrate medications up slowly, use molecules that have a potential to address more than one symptom, and avoid polypharmacy. Combining pharmacological and non-pharmacological approaches to address specific symptoms is important. For example, gait difficulties can be addressed with physical therapy (with a focus on improving ataxia or muscle strength depending on presentation), spasticity management (stretching, anti-spasticity medications, botulinum toxin injections, baclofen pump), and fatigue with dalfampridine. A significant emphasis should also be placed on physical, occupational, and speech therapy. Evaluation and treatment by a multi-disciplinary team is key to provide optimal care across the range of dysfunction in progressive MS. General wellness measures and management of comorbidities should always be discussed with patients, most importantly hyperlipidemia, hypertension, and diabetes control, consuming a healthy diet, weight loss, smoking cessation, vitamin D supplementation, osteoporosis management, and emotional wellness [159].

## 7. Challenges in Progressive MS Treatment and Research

There are many unmet needs in the field of progressive MS. First, our experience with anti-inflammatory medications like ocrelizumab and siponimod has shown positive but modest results. These medications work on the inflammatory component of the disease, and their potential mechanism of action on the neurodegenerative aspect of MS is probably minimal, if any. As discussed above, the rituximab/ocrelizumab and fingolimod/siponimod experiences in progressive MS provide evidence that therapeutic approaches for progressive MS should probably focus on a different pathophysiological aspect of the disease. Second, trial methodology has significant implications for the effect of agents in progressive MS. Study population selection and amount of disease activity (pre-trial and in-trial annualized relapse rates, presence or absence of baseline gadolinium enhancing lesions) in enrolled subjects are key in driving these efficacy differences between therapies. The definition of progressive MS also varies between trials, and often but not always, a minimum confirmed EDSS step of three at trial entry is required in progressive MS trials, which makes the study populations somewhat heterogeneous and trial results inconsistent and difficult to compare across studies [57].

Third, the selection of appropriate clinical outcome measures plays an important role in capturing treatment effects in progressive MS trials, as discussed earlier in this manuscript [160]. Non-ambulatory patients are typically excluded from progressive MS trials, and the benefit of different therapies on functional domains other than gait function could yield more promising results, specifically in this patient population. For example, studying hand and cognitive function or using composite outcomes like the MSFC rather than relying solely on ambulation as a primary endpoint could inform treatment effects in a more sensitive way. Hand function might be more amenable to treatment compared to lower extremity function in patients with more advanced disability. In the ASCEND trial for instance, natalizumab was associated with a 44% reduction in the relative risk of confirmed upper limb disability progression measured by the 9HPT (adjusted OR 0·56 (95% CI 0·40–0·80); *p* = 0·001), whereas no benefit was observed on other measures of disability like the EDSS and the T25FW [101]. In a pre-specified baseline subgroup analysis of patients with EDSS ≥6.0 and age >45 years from the OROTARIO trial, ocrelizumab also reduced disability progression as measured by the 12-week confirmed 9HPT in older non-ambulatory patients [161]. Moreover, despite the high prevalence of cognitive dysfunction in progressive MS, there are methodological gaps in outcome measures of cognition in MS, and cognitive function is not adequately and comprehensively evaluated in trials [57,162]. The use of composite outcome measures has been used increasingly in progressive MS trials, and appear to have higher sensitivity to changes. For example, the primary endpoint of the INFORMS trial was a novel composite outcome measure that was defined as a 3-month confirmed change from baseline of the EDSS, the T25FW, or the 9HPT, and although the trial was negative, this outcome measure detected changes with excellent sensitivity in this population [77] Trials evaluating therapies with a remyelinating or repair potential are expected to have a modest clinical effect and can particularly benefit from using composite measures to enhance detection of changes. This approach was used in the opicinimab (SYNERGY) [129] and ibudilast (SPRINT-MS) [132] trials. MRI outcome measures also have some limitations. For example, measures of whole brain atrophy are mostly used as primary endpoints, and have the issue of being variable, which is a concern for interpretability. Other measures like cortical atrophy or magnetization transfer ratio might be more useful depending on the population and mechanism of action of the investigational product.

Finally, the efficacy of molecules with a potential for remyelination and/or neuroprotection needs to be confirmed in large trials with clinically meaningful outcome measures, which will require time and resources. Ultimately, for FDA approval, an agent that can demonstrate clinically meaningful changes in disability measures in large multicenter phase 3 trials is needed. To be able to achieve that, research on the optimal outcome markers is needed. Moreover, many of these molecules are old drugs that do not generate interest in the pharmaceutical industry.

Overall, optimal and novel trial methodology and development of sensitive and clinically meaningful outcome measures and biomarkers are needed in the near-future. The International Progressive MS Alliance and the UK Expert Consortium for Progression in MS Clinical Trials have a mission to expedite development of therapies for progressive forms of MS, which includes the development of optimal trial designs and more responsive outcome measures.

## 8. Conclusions

A significant amount of effort is delivered to improve knowledge in the field of progressive MS. Future trials will incorporate lessons from previous trials, and hopefully, therapies that halt or even stop neurodegeneration in MS will be available in the future.

## Figures and Tables

**Table 1:** Potential remyelinating and neuroprotective molecules studied in multiple sclerosis.

Therapy	Potential Mechanism of Action	Trials	Primary Endpoint	Results
Remyelination strategies				
Clemastine fumarate	First-generation anti-histamine, promotes remyelination and oligodendrocyte differentiation via anti-muscarinic effect [123,124]	ReBUILD [125]	Shortening of P100 latency delay on visual-evoked potentials at 150 days	Improvement in P100 latency of 1.7 ms/eye (95% CI 0.5 to 2.9, *p* = 0.0048) with clemastine
Opicinumab	Anti-LINGO-1 antibody, promotes remyelination and oligodendrocyte differentiation via blocking of inhibitory adhesion molecule [126,127]	RENEW [128] (in acute unilateral optic neuritis)	24-week change in optic nerve conduction latency using full-field visual evoked potential	Non-significant trend towards improvement in the intention-to-treat analysis, modest but significant benefit at week 32 in the per-protocol analysis
		SYNERGY [129,130] (in RRMS and SPMS with active disease)	Percentage of participants with ≥3 month confirmed improvement of composite endpoint (EDSS, T25FW, 9HPT, PASAT) over 72 weeks	Benefit seen in those receiving the 30 mg/kg dose.
Neuroprotection strategies				
Ibudilast	Phosphodiesterase-inhibitor, inhibits macrophage migration inhibitory factor, and toll-like receptor 4 [131]	SPRINT-MS [132]	Progression of whole brain atrophy over 96 weeks	48% slowing in the rate of atrophy progression with ibudilast compared to placebo
Simvastatin	HMG-CoA reductase inhibitor, inhibit MHCII-restricted antigen presentation, shifts cytokine production from a pro- to an anti-inflammatory response, decreases T-cell proliferation [133]	MS-STAT [134]	Progression of whole-brain atrophy, change in EDSS and total MS Impact Scale-29 at 24 months	Decrease in annualized rate of whole brain atrophy compared to placebo, benefit on EDSS and MS Impact Scale-29 as well
Lipoic acid	Endogenous antioxidant, various potential mechanisms, including free radical scavenging, oxidative damage repair, downregulation of inflammatory cytokines, T-cell migration in CNS inhibition [135,136]	Spain et al. [137].	Annual percent change of brain volume	68% reduction in the rate of brain atrophy compared to placebo over 24 months
Phenytoin	Selective sodium-channel inhibitor, reverses sodium influx, which drives calcium influx via reverse operation of the sodium/calcium exchanger after axonal injury [138]	Raftopoulos et al. [139].	RNFL thickness in the affected eye	30% reduction in the extent of RNFL loss with phenytoin compared with placebo at 6 months
Mesenchymal stem cells *	Pluripotent non-hematopoietic precursor cells (isolated from bone marrow or adipose tissue), release of soluble trophic factors that promote intrinsic tissue repair mechanisms [140]	Multiple small clinical trials and open label studies using variable route of administration and dosing regimens [141,142,143,144,145,146,147,148,149]	Variable endpoints depending on trial	Good safety and tolerability, efficacy not yet established [150]
		Phase II randomized, double-blind trial, MESEMS (NCT01854957) [151]	Safety, reduction in the total number of contrast-gadolinium enhancing lesions	Ongoing
		Open-label study, MSC-NTF Cells (NCT03799718)	Safety, T25FW change from baseline, changes in neurotrophic factors	Ongoing
High-dose biotin (MD 1003) *	Essential co-factor for five carboxylases involved in fatty acid synthesis and energy production, promotes remyelination, and reduces axonal hypoxia [152]	Sedel et al. (pilot study) [153]	Shortening of P100 latency on visual-evoked potentials	Improvement or normalization of P100 latency
		Tourbah et al. (randomized, double-blind placebo-controlled trial) [154]	Proportion of patients with disability reversal on EDSS or T25FW at month 9, confirmed at month 12	2.6% of treated patients achieved the primary endpoint versus none of the placebo-treated patients (*p* = 0.005)
		Birnbaum et al. (open-label study of compound medication, not MD 1003) [155]	EDSS worsening or improvement while on treatment (3 to 12 months)	No benefits observed

* possibly dual effect on remyelination and neuroprotection. 9HPT: 9-hole-peg test, EDSS: Expanded disability status scale, LINGO-1: Leucine-rich repeat and immunoglobulin domain-containing neurite outgrowth inhibitor receptor interacting protein-1, PASAT: Paced auditory serial addition test, RRMS: Relapsing–remitting multiple sclerosis, RNFL: Retinal nerve fiber layer, SPMS: Secondary progressive multiple sclerosis, T25FW: Timed-25-foot walk.
